# A Pain in the Butt: A Case Series of Gluteal Compartment Syndrome

**DOI:** 10.5811/cpcem.2021.3.51801

**Published:** 2021-04-19

**Authors:** Jessica Ray Jackson, Kraftin Schreyer

**Affiliations:** Temple University Hospital, Department of Emergency Medicine, Philadelphia, Pennsylvania

**Keywords:** Gluteal compartment syndrome, fentanyl

## Abstract

**Introduction:**

Gluteal compartment syndrome is a rare and difficult-to-diagnose form of compartment syndrome.

**Case Series:**

We present three patients with gluteal compartment syndrome and review the clinical presentation, imaging, and laboratory findings that assist in diagnosis. Suggestions for more readily diagnosing gluteal compartment syndrome are provided.

**Conclusion:**

Emergency physicians must be familiar with the diagnosis and management of gluteal compartment syndrome to prevent the significant associated morbidity and mortality.

## INTRODUCTION

Compartment syndrome occurs when increased pressure in a closed fascial area leads to decreased tissue perfusion. Gluteal compartment syndrome (GCS) is a rare form of compartment syndrome that is difficult to diagnose and lacks clear diagnostic guidelines.^1^ Any of the three gluteal compartments, the anterior tensor fascia lata compartment, the gluteus medius and minimus compartment, or the posterior gluteus maximus compartment, can be affected.^2^ Approximately 50% of cases are due to prolonged immobilization secondary to surgery, or alcohol or substance use.^3^ Benns et al showed that compartment syndrome in patients with opioid use disorder (OUD) was more likely to be gluteal (31.8%) when compared to other etiologies and led to prolonged hospital admissions.

Delays in seeking care and in diagnosis have been shown to lead to complications including tissue loss in 27% of patients, the need for hemodialysis in 36.4% of patients, and residual motor or sensory deficits.^3^,^4^ One series found that patients treated with surgery had no subsequent neurologic deficits, while all patients managed conservatively had some residual deficit.^5^ Therefore, GCS should be considered a surgical emergency.

As a significant proportion of cases are related to intoxication, diagnosis is often complicated by a limited history and exam. Patients may have altered mental status prior to presentation or may present to the emergency department (ED) obtunded. Aside from elevated creatine kinase (CK), GCS can lead to several lab derangements that may aid in diagnosis. Both alanine aminotransferase (ALT) and aspartate aminotransferase (AST) are found in skeletal muscle, although ALT is more specific for the liver. As a result, compartment syndrome may lead to elevated AST and ALT, which can be incorrectly interpreted as hepatic dysfunction.^6^ Similarly, patients with rhabdomyolysis have shown to have a false-positive troponin rate of 17%, without any association with cocaine use or renal failure.^7^ Patients may also have elevated creatinine and potassium.

The following cases series includes three patients who developed GCS in the setting of substance use, likely secondary to prolonged immobilization. All patients presented to the ED of the Episcopal Campus of Temple University Health System, a community affiliate located in the Kensington section of Philadelphia, Pennsylvania. In 2018, Kensington saw the greatest number of nonfatal overdoses and the highest number of naloxone administrations in Philadelphia.^8^,^9^ In 2019, 13.5% of the 46,000 annual visits to the Episcopal ED were related to OUD. Episcopal Hospital has an associated crisis response center (CRC) that provides 24-hour psychiatric emergency services; however, consultative services, including surgery, are not available. Patients who need emergent surgical evaluation are transferred to the main academic center. All patients in this series were diagnosed clinically without measurement of compartment pressures.

## CASE SERIES

### Case 1

Case 1 was a 38-year-old female with past medical history (PMH) of substance use disorder (SUD) who presented in January 2020 with altered mental status. The patient complained of right shoulder pain but was minimally cooperative due to suspected phencyclidine intoxication. A radiograph of her right shoulder did not show any acute pathology, and she was discharged. She later presented to the CRC where she was placed in observation due to concern for intoxication and was discharged after two hours. She returned to the ED one hour later via emergency medical services (EMS) with weakness and difficulty walking after being found outside. Her initial vital signs were as follows: 147/106 millimeters of mercury (mm Hg); heart rate 95 beats per minute; respiration rate 20 breaths per minute; oxygen saturation of 100% on room air; and initial temperature less than 80º Fahrenheit. She received multiple doses of lorazepam for agitation, and continued to complain of muscle cramping and right shoulder pain. Initial laboratory results were hemolyzed.

Repeat laboratory studies after initiation of intravenous fluids were notable for the following: creatinine 3.77 milligrams per deciliter (mg/dL) (reference range 0.90–1.30 mg/dL); potassium 5.6 millimoles per liter (mmol/L) (3.5–5.2 mmol/L); ALT 2440 units per liter (U/L) (0–44 U/L); AST 7087 U/L (0–34 U/L); troponin 4.1 nanograms per milliliter (ng/mL) (0.00–0.10 ng/mL); CK 296,865 U/L (49–174 U/L); and a urine drug screen (UDS) positive for fentanyl, cocaine, and phencyclidine. The elevated CK prompted reassessment and she was found to have a firm right shoulder and left buttock without associated skin changes. This was best appreciated while the patient was standing, which revealed an obvious difference in the size of the right and left buttocks. When prone, her gluteal compartments were not notably firm.

She was then transferred emergently for surgery evaluation, and a computed tomography (CT) series was performed. The CT chest showed right lateral chest wall edema and possible aspiration; the CT abdomen and pelvis showed a collection in her left gluteal musculature representing hematoma or abscess, and intermuscular edema along the anterior and posterior compartments of the left thigh. Ultimately, she was taken to the operating room (OR) for right shoulder fasciotomy. On hospital day three, she was taken back to the OR for left gluteal fasciotomy and was started on hemodialysis. She later had renal recovery and was able to discontinue hemodialysis prior to discharge on hospital day 25.

**CPC-EM Capsule**

What do we already know about this clinical entity?

*Gluteal compartment syndrome (GCS) is rare and difficult to diagnose. About 50% of cases are due to prolonged immobilization secondary to surgery, or alcohol or substance use*.

What makes this presentation of disease reportable?

*This case series demonstrates the importance of labs and of examining patients while they are standing in order to diagnose GCS, as well as the association of GCS with fentanyl use*.

What is the major learning point?

*Providers must keep a high index of suspicion for GCS, particularly in patients who use fentanyl*.

How might this improve emergency medicine practice?

*More rapid diagnosis of gluteal compartment syndrome can help prevent subsequent morbidity and mortality*.

### Case 2

Case 2 was a 37-year-old male with a PMH of SUD who presented to the ED in June 2020. He had reportedly been given naloxone from EMS after using opioids and reported not feeling well. He was uncooperative and shouting on arrival and complained of possible assault, noting right hip and back pain. His initial creatinine was 4.33 mg/dL and potassium was 6.9 mmol/L, which prompted reassessment for compartment syndrome. Examination with the patient standing showed edema and erythema of the right buttock. The left buttock, although less edematous, was also tense. Given those findings, he was transferred for emergent surgery evaluation. Additional laboratory studies included a CK of 218,142 U/L and a UDS positive for fentanyl, cocaine, cannabinoids, and amphetamines. He subsequently had CT imaging of his head, chest, abdomen and pelvis, which was remarkable for left lateral gluteal and pelvic musculature swelling, as well as edema of the right anterior abdominal musculature and right gluteus that were presumed to be secondary to blunt injury. He was taken to the OR that evening for left gluteal fasciotomy and was started on hemodialysis. On hospital day two, his right buttock was noted to be firm and he was taken back to the OR for right-sided gluteal fasciotomy. He also had renal recovery and was discharged on hospital day 18.

### Case 3

Case 3 presented to the ED as an unidentified male in July 2020. He was subsequently identified as a 34-year-old with a PMH of SUD and schizophrenia. He was brought in by EMS after being found unresponsive on the sidewalk and was given naloxone prior to arrival. On arrival to the ED, he had a Glasgow Coma Scale of seven with increased work of breathing. He was intubated for airway protection and shortly after was found to have elevated creatinine of 1.81 mg/dL and potassium of 6.7 mmol/L. This prompted a repeat examination and he was found to have a firm right buttock without skin findings. His other labs subsequently resulted and were remarkable for ALT 114 U/L, AST 205 U/L, troponin 0.31 ng/mL, CK 16,854 U/L, and UDS was positive for fentanyl, cocaine, cannabinoids, phencyclidine, and benzodiazepines.

He underwent CT imaging prior to transfer to assess for any traumatic injuries. His CT head was notable for sulcal effacement, ventricular narrowing, and multifocal hypoattenuation likely acute infarction. The CT chest showed nodular and consolidative ground glass opacities; CT abdomen and pelvis showed right buttock with large expansile phlegmon, and ultrasound was recommended to assess for drainable collection. Shortly after transfer he became hypoxic from worsening pulmonary infiltrates consistent with acute respiratory distress syndrome. He then suffered cardiac arrest but achieved return of spontaneous circulation after multiple rounds of cardiopulmonary resuscitation. Due to critical illness, he had a beside gluteal fasciotomy. Unfortunately, he was found to have catastrophic anoxic brain injury. The family agreed to terminal extubation on hospital day six. He did not require hemodialysis.

## DISCUSSION

Although rare, GCS may be the most common form of opioid-related compartment syndrome. With the national increase in OUD, providers must be vigilant for this entity. Each patient in this case series had multiple substances on their UDS and, notably, all were positive for fentanyl. Compared to other opioids, fentanyl is more potent and is known to cause rapid respiratory depression, unconsciousness, and muscle rigidity.^10^ This combination may have contributed to the development of compartment syndrome in these patients. While GCS has been linked to OUD, we have not seen previous documentation of the relationship with fentanyl specifically.

Compartment pressures can be used in the diagnosis of GCS but may be difficult to obtain. Most authors suggest an intracompartmental pressure of 30 mm Hg as the threshold for initiating treatment, but GCS is ultimately a clinical diagnosis.^11^ Patients who are unable to give a reliable history or participate in an exam are even more difficult to diagnose. We suggest having patients stand to more easily assess the gluteal compartments and compare contralateral edema. The deeper gluteus medius and minimus compartment is frequently affected. Compartment syndrome may not be detected if the overlying gluteus maximus is soft, and the patient is lying prone.

No patients in this case series were found to have traumatic injuries, as determined by CT, nor signs of injection drug use in the affected areas on examination. The areas that were later identified as compartment syndrome were described as either hematoma or abscess based on CT findings. Providers should be aware that GCS cannot be diagnosed based on imaging findings and may appear infectious or post-traumatic.

Of the two patients who had hepatic function tests and troponin performed, all levels were elevated with AST higher than ALT. Neither was determined to have a primary hepatobiliary or cardiac issue. All three patients had elevated creatinine and potassium, which may be the first clue to the diagnosis and should prompt reassessment of all compartments. While an elevated CK should also trigger reassessment, the laboratory may have to perform serial dilutions, which could delay reporting of the result. In this series, Case 2 and Case 3 were both transferred for surgical evaluation before their CK resulted.

## CONCLUSION

This case series shows that multiple deleterious outcomes can be associated with gluteal compartment syndrome. It further highlights the challenges in diagnosing GCS and its association with OUD, particularly with fentanyl use. Providers must maintain a high index of suspicion with early recognition of a constellation of abnormal laboratory values and thorough physical examination to prevent subsequent morbidity and mortality in patients who may be unable to assist in diagnosis.
